# Traumatic chain: Korean–American immigrants’ transgenerational language and racial trauma in *Native Speaker*

**DOI:** 10.3389/fpsyg.2022.912519

**Published:** 2022-11-04

**Authors:** Muhammad Sohail Ahmad, Shazmeen Nawaz, Zainab Bukhari, Mubashar Nadeem, Rana Yassir Hussain

**Affiliations:** ^1^Department of English, Division of Arts and Social Sciences, University of Education, Lahore, Pakistan; ^2^Department of English, Govt. Associate College for Women, Quaidabad, Pakistan; ^3^UE Business School, Division of Management and Administrative Science, University of Education, Lahore, Pakistan

**Keywords:** Korean immigrants, language vulnerability, racism, trauma, identity crisis

## Abstract

The premise of this study is to look at the intergenerational transferal of language and racial trauma of Asian immigrants in general and Korean–American immigrants in particular to a western country, the United States of America. This study investigates trauma from a psychological standpoint, based on Chang-Rae Lee’s novel Native Speaker. In describing a marker of citizenship, the novel’s title also points to who is the native language speaker and who is a native of a country, and why one who is not may be excluded. The Korean immigrants’ vulnerability to the English language and racial differences highlights their status as minority “others,” and they suffer from transgenerational trauma. As a result of transgenerational traumatic effects, Henry (the protagonist) has various traumatic side effects such as dysphasia, aphasia, and parasomnia and finally leaves no stone unturned to recuperate from trauma. The Trauma of the Unspeakable theory by Michelle Balaev is used in this article to show how trauma affects people’s minds.

## Introduction

A glimpse of the history of immigration in America reflects that the movement of Asians to America is always tinted by the deliberate interests of the controlling powerful group. Kingston (1981) uses the railroad as a significant symbol to narrate the story of Chinese–American history in *China Men*, whereas Chin (1988) uses the *railroad* as a significant symbol to narrate the story of Chinese–American history in the short stories “*Eat and Run Midnight* People” and “*Railroad Standard Time*.” However, the civil war changed the position of Asian migrants, as historians Okihiro and Jung (2014) claim that Asian laborers became a great alternative to Afro–Americans after liberation and have been molded by selective minority “successes” discourse to organize veracious Afro–Americans during the fiery movement of civil rights in the 1960s in the south. Hence, this squeezing of Asian labor entertained the political and economic hegemony of Whites but ended up because Whites visualized it as a gruesome threat to the cultural and racial “purity” of White America. Thus, Whites, according to Robert (1999), started to regard these Asian immigrants as “anomalies” that “broke the chain of westward historical progress” and “pollutants in the symbolic structure of society.”

Daniels and Kitano (1988) divide the history of Korean immigrants to America into three waves. In 1903, the “first wave” begins with the immigration of 7,000 Korean laborers, whereas the rupture of the Korean War (1950–1953) constitutes the “second wave” of immigration in which orphans, students, and wives of American soldiers build on the number of Koreans in the United States. The post-1965 period contains a “third wave” of immigration. The influx of Korean immigrants to American soil increased considerably, especially in Los Angeles, albeit their hopes were shattered when they realized the difference between the beautifully imagined country and its actual ugly picture after their arrival, making them unable to adjust.

Looking critically, it is sufficing to say that the American environs robustly encourage immigrants to “shed” their identities in America, which Lim (1992) posits as “‘American’ qualities” that “collapse the diasporic subject into the amnesiac condition of the “new American” hybridity and demand assimilation.” However, Hwangbo (2004) calls this assimilation the sacrifices of certain ethnic groups on American soil that are not “good enough,” otherwise labeled as “alien.” Among these ethnic groups, Asian–Americans endure the agonies of the devoted assimilation-centered rhetoric along with the subdued status of “others” that, for Betz (2019), is a “North American phenomenon.” Moreover, researchers (Kim and Kang, 2002; Betz, 2017) argue that mainstream American society sees Asian–Americans as foreigners and sojourners, regardless of their several generations–long histories of settlement and associate this ideology with “anti-immigrant prejudice and hostility” where Asian–Americans, to White Americans, are “small and dark” people who “huddle over dishes of strange food.” For Betz and Meret (2009), both sides considered each other rigid, fundamental, and not ready to accept the other culture and go through an assimilation process that made Asian immigrants and migrants totally different from West.

The treatment of hegemonic patterns with Asian–Americans varies from that of Afro–Americans (no matter whether they are both exploited based on the racial discrimination) and the status of Afro–Americans as Americans is not challenged as Asian. Asian immigrants undergo angst of continuous impediments and retro movements in their assimilation process, whereas the graph of assimilation of European immigrants is very smooth. As a social psychologist (Markus, 2010), he deconstructs and connects the cultural crisis of immigrants as two key processes that are not so much “I think, therefore I am,” but rather “you think, therefore I am”; second, Markus challenges the role of identity by saying “we (immigrants) do not see things as they are, we see them as we are.” Similarly, a Norwegian report (2017) shows that “high immigration” raised the concern that “increased cultural and religious diversity may affect social trust and unity in the country.” Asian–Americans are thought to be rigid, fundamental, and not ready to accept other cultures. They also have to go through an assimilation process that makes them very different from African Americans, who were easy to assimilate. As a result, they experience anxiety and depression and may have a detrimental influence on health and psychopathology issues (van der Kolk et al., 2012).

Along with historical background, the 2 t century saw a rise in racism against Asian immigrants in America that Togral (2016) calls a superiority over Asian ethnic minority people based on color, nationality, or language and a danger to everyday lives. However, Fekete (2009) called it xeno-racism and Friedman (2017) characterized it as “xenophobic nationalism,” where xeno-racists or nationalists perceive ethnic groups as inferior human beings or regard them as dangerous people to the host nation and consider all non-born as a menace. Racism and prejudice are the underlying causes of blind and illogical hatred. Recent events have seen notable instances of racism against Asian immigrants in America. For instance, Brockell (2021) stated that anti-Asian racism is “a serious problem” where Asians, especially Koreans, are being called “China Virus,” which is “racist and creates xenophobia” to call Asian immigrants “China Virus” (Chiu, 2020). In another incident, at three Atlanta-area spas, a gunman killed eight people, including six Asian–American women (Fausset et al., 2021). Taking pride in belonging to a particular nation is innate, but discrimination on this basis to increase authority is unethical and often results in silencing the survivor and causes a drastic identity change, which psychologist Harvey (2002) calls a fundamental loss. The victims of trauma do not mourn their pain in front of anyone and need language to recuperate their wounds. Yet it scares them the most because the trauma impacts identity and the victims’ linguistic repertoire, i.e., their tendency to learn the language, retain, abandon, or use a specific language, or their will to seek escape from traumatic memory by silencing themselves (Kanwal, 2015; Humaira, 2017; Langah, 2019). To answer the questions, the authors figure out how language, silence, and trauma are connected through textual analysis of the novel *Native Speaker* by employing Balaev’s theory of Trauma of the Unspeakable.

The Trauma of the Unspeakable is the primary form of language trauma for Korean immigrants and for Balaev (2012) is a “speechless fright” that destroys the victims’ identity. Furthermore, Balaev (2008) added that if traumatized people do not speak out and accept such events as a necessary part of their existence, they will constantly be stuck within the “black hole” of trauma that saps their vitality by fixating on the trauma. Likewise, Caruth (1996) asserts that the unspeakable nature of trauma and the intensity of its experience can severely impact the psyche and cause everlasting harm to the victim’s identity as trauma resides in the unconscious mind in a timeless and wordless state that causes continuous pain and damage to the psyche.

The investigation of any imbroglio in the cross-cultural text, such as mourning, trauma, and loss, should consider the significance of the socio-political background that causes a sense of loss related to migration and delays mourning. This manuscript addresses the psycho-social aspect of trauma with reference to the problems of social despotism as faced by Korean–American immigrants in an American community by presenting George Washington Park–the protagonist’s father, Henry–the protagonist, Henry’s son–Mitt, and the minor characters–Kwang (a politician) and Ajuma (a housemaid in Henry’s house). George Washington Park endures racism helplessly and silently due to the “loss” of language, being deprived of the ability to speak his rights and fight against racist violence. Shame and forced silence make him invest in the belief that removing linguistic and ethnic disparities through total assimilation would save his family by insisting his son speak only English and get a “white” education. Witnessing his father’s humiliation and living with the news of “another cabbie (’s)” death (Lee, 1995) creates what Brown (1995) refers to as “insidious trauma” for Henry, who is unable to detach himself from other “strangers from a different shore,” as Taki (1989) refers to them, no matter how hard he tries to become a native speaker of English and build an “American” family with a white wife and child. He feels like “a trapped animal” who cannot escape a cage (Lee, 1995). This belies the problematic entanglement of language and race that Asian immigrants and their descendants experience regardless of their language proficiency. Ludwig (2007) says, “the way you speak defines you.” It resonates differently for Asian–Americans, “regardless of how much Asian–Americans are entrenched in American society, they nevertheless experience ambiguity and confusion” (p. 15). In addition to the elderly, Mitt, the child from the third generation, also endures the transgenerational trauma of silence at an earlier age. The racism that has resulted in recent anti-Asian hate crimes testifies to the root cause that frames Asian–Americans as perennial foreigners.

## Literature review

According to Alexander et al. (2004), cultural immigration or relocation entails separation, anxiety, and an inexorable loss of identity. In this regard, various psychoanalytic theories, such as self-psychology by Kohut (1971), object relation theory by Winnicott (1965), and that of psychoanalyst Antokoletz Juana (1993), while sociologist (Alexander et al., 2004), shed light on the cross-cultural intricacies faced by immigrants as a result of their passage to the host country, in the form of the loss of their native culture and identity. Even though Antokoltz links the pressure of forced assimilation and false self-consciousness, he leaves a gap in which the group-specific traits related to false self-image become transparent in a coded way under the burden of assimilation, defining the historically rooted, and socially constructed experiences of that very group.

Owing to these experiences of adjusting to a foreign land, the individual endures panic anxiety and racial insecurity based on the ontological grounds. Grinberg and Grinberg (2004) argue the “subject’s reactions are not always expressed or visible” (p. 12) and often delay their mourning, thus developing a “latency period” as found in cases of trauma that Freud has already discussed and developed the labyrinthine connection between cultural displacement, trauma, its transference from one generation to the other, and the return of the suppressed life in Moses and Monotheism that Caruth (1996) posits “the story of the way in which one’s own trauma is tied up with the trauma of another” (p. 8), as is the case in the novel *Native Speaker*.

In the field of humanities, along with psychologists, researchers have highlighted the psychological trauma of Korean immigrants and have also attempted to read the phenomenon of Korean immigrants and identity crises from the perspective of political discourse (Chen, 2002; Huang, 2006; Kasinitz, 2007; Kim, 2009; Vinu, 2019), while others studied it through the lens of a particular Korean culture and its effects on identity (Miller, 2016; Hanifa et al., 2019), albeit failing to show and relate with the concept of transgenerational trauma. Literature has also been read using the same lens of Orient/Occident as well as from the angle of mistreatment of diasporic Asian–American or Korean immigrants (Zhou, 1997; Kim, 2008; Ninh, 2011; Sung, 2018).

Working from a psychologist’s perspective, the current debate on Korean American identity in Korean literature seems to have missed the contribution of the psychoanalytical approach toward the understanding of intergenerational trauma and identity crises (Arac, 2009; Markus, 2010). For example, Markus deconstructs and connects the identity crisis of Korean immigrants as two key processes that are not so much “I think, therefore I am,” but rather “you think, therefore I am.” Second, Markus challenges the role of identity by saying “we (immigrants) do not see things as they are, we see them as we are.” Although Markus attempts to study Korean immigrants’ diasporic identity issues by linking them with identity issues, her research does not deal with the role and contribution of literary theory to the understanding of intergenerational trauma. Similarly, Arac studies racial melancholia and argues that immigration and its demanded assimilation are a trauma for Asian–Americans and others. By doing so, he misses out on exploring the Korean immigrants’ language trauma with respect to intergenerational exchange.

In an effort to fill this gap by employing the concept of The Trauma of Unspeakable in the novel *Native Speaker* by Chang-Rae Lee, this study highlights why Korean American immigrants suffer from inherited loss and suffer from transgenerational trauma during their stay in America. The research also shows how the loss of a language (Korean/American) and traumatic suffering from it changes victims’ personalities and compels them to feel in limbo. Although different researchers have focused on the humanitarian crises and suffering of Korean immigrants from a political perspective, the intergenerational trauma from a psychoanalytical perspective did not get the attention it deserves the most. To the best of researchers’ knowledge, no researcher has analyzed the selected novel to highlight language trauma by applying the psychological approach of Michelle Balaev’s theory of The Trauma of Unspeakable and identity. To address these questions and issues, the researcher has divided the manuscript into three sections: cause, effects, and recuperation from trauma.

## Methodology

The research is qualitative, and the researchers aim to utilizing the “text and context of an event” (Alexander et al., 2004) to examine its treatment and determine the causes of psychological trauma. The study uses a descriptive method to analyze and describe the selected text. This manuscript draws its methodology from the transgenerational trauma theory of Balaev (2008) and focuses on the notion of transgenerational trauma with reference to an event by heavily relying on issues of language and racism to examine the selected novel. The concept of transgenerational trauma refers to or occurs at the individual level, which, in Alexander’s (2013) words, is subjected to dreadful events and is different from the communal trauma presented by Smelser. Now turning to Freud’s writing (which is deemed necessary for the implication of individual trauma) initially focused on the physical trauma related to hysteria, cause, development, and effects.

According to Freud (1961), from a psychological perspective, trauma is a shock that causes a “breach” in the “protective barrier” of the mind at an individual level, whereas Žižek (1989) calls it a shock that interrupts the “symbolic mechanisms” and destroys the stability of the subject’s symbolic world. However, Allen (1995) posits that trauma is “a feeling of utter helplessness.” Regarding the cause and experience, Freud (1961), in his work *The Study of Hysteria*, highlights “a passive sexual experience before puberty,” usually a molestation or seduction experience caused by an “event,” which brings a feeling of horror, fear, shame, and suppression in the unconscious. The development of anxiety is associated with a status of latency or incubation. Freud reiterates that people suffering from mental anxiety are held captive to painful experiences of their distant past, and he characterized the memory of trauma as “a foreign body which long after its entry must continue to be regarded as an agent that is still at work” (Breuer and Freud, 1955). The memory is desperately clinging to the past because it has significant value, and this fixation can last a lifetime. Also, it is worth noting that Kardiner (1941) agreed with Freud that the “nature of re-enactment” allows people to reprogram their minds as regards how they would have or should have responded to the original events, and as he also pointed out, “the subject acts as if the original traumatic situation was still in existence and engages in protective devices that failed on the original occasion” (p. 82). Furthermore, Freud extends his idea that the negative effects are a condition resulting from traumatizing events and characterized by the re-enactment of these events in unwanted dreams, flashbacks, recurring thoughts, and accidental encounters with sensory fragments in daily life associated with the original traumatic experience and events.

Michelle Balaev also focuses on (cause, effects, and recovery) individual psychological trauma concerning traumatic experiences by extending Freud’s concept of traumatic experience and memory. Regarding cause, Balaev (2008) argues that the terrifying “intimately personal experience,” for instance, racism or language barriers of Asian immigrants, organizes the memory and meaning of trauma with a “temporal gap” that Tal (1996) in *Worlds of Hurt* calls “normal conception,” and such a temporal gap is only achievable through the actual representation or narrative call of traumatic experience. The narration of such traumatic experiences can disturb consciousness and be transferred “through verbal or written acts of remembering” (Balaev, 2008) from traumatized people to non-traumatized individuals intergenerationally. The cause of racism shows intergenerational traumatic experiences focus on the personal loss (loss of language in this article) experienced by an individual. Personal loss is defined as the living or first-hand experience of a traumatic event that is “created” rather than “born,” according to Alexander et al. (2004). The theory of intergenerational trauma also says that a person’s current identity can be “vicariously traumatized” or, in Bouson’s model, “intergenerationally transmitted” because of shared ancestry or traumatic experiences.

Paying attention to effects, unspeakable tropes, and silence, the theory also provides a concept regarding the formation of identity concerning the intergenerational sharing of “loss and suffering” because the actual event is transmitted to descendants of the same racial, ethnic, religious, or gender group (Balaev, 2008). Similarly, clinical psychologist Brown (1995) argues that loss and “traumatic effects of oppression” show no sign on the body at a given moment but leave a long-lasting effect on the psyche and identity of the next generation. In line with Balaev and Brown, Erikson (1963), an American–German psychologist, also took a member of an ethnic group who, like his elders, “knows his (lowest) place” in White-dominant society and remains silent. His silence would link him with the Trauma of the Unspeakable. By taking the views of Michelle Balaev on the unspeakable trope, Caruth (1996) viewed the unspeakable from the perspective of “cultural values” that break or tear up the victim’s self-narrative. Thus, the victim finds expression in violent acts, dissociation of self, or unemotional deadening of self.

The trope of the unspeakable as silence is employed as another means to show the effects of an intergenerational traumatic experience by enhancing its horrible intensity and “speechless fright that destroys the identity” (Balaev, 2008). Considering the experience and memory, the unspeakable or silent nature of trauma and the intensity of the experience behind it can severely impact language and awareness. It causes permanent harm and necessitates extraordinary narrative skills to find expression for suppressed feelings. Finally, the model’s definition shows that once the trauma is “spoken,” it no longer remains “unspeakable” or “traumatic.” Hence, the more a victim speaks or repeats this process of speaking, the more he gets better. If the traumatized person fails to speak, he will remain in the “black hole” of trauma that saps his vitality by fixating on the trauma (Caruth, 1996). So, any attempt to get back on his feet and re-establish himself in society is doomed to fail because no one can say the right thing until the traumatic experience is said in a way that makes sense.

Keeping in view this scholarship of theorists, the research aims to study the construction of “trauma of the unspeakable” in comparison with the selected literary texts and to know the ways that, in Balaev’s words, it is transmitted “intergenerationally.” This manuscript focuses on multiple key points of Balaev’s work: traumatic experience; memory and its connection to previous generations; the effects on people; and the formation of a new identity.

## Results

The authors argue that traumatized people suffer from “not known” trauma that refers to/occurs at an individual level and can also be connected with Freud’s (1961) term “traumatic neurosis” and “factor of freight” (p. 32). In this situation, fear usually comes out through the re-enactment of traumatic events that the patient “reproduces not as a memory but as an action; he repeats it, of course, without knowing that he is repeating” (Freud, 1961) in unwanted dreams, like Henry in the book, where it is unknown and lives in the unconscious, but later comes back to consciousness and comes to life.

Keeping in view these interpretations of psychological trauma concerning traumatic events, the study examines and posits, in response to Balaev’s concept of trauma and dream, that a victim is unable to experience the trauma fully on the spot because (Caruth, 1996) it is “too soon, too unexpectedly, to be fully unknown.” However, the victim repeats the events and may carry on struggling to come to terms with the experience that is behind the repetition. Here, the basic diagnosis of trauma from disastrous incidents does not include the complication of “unassimilated nature,” which is unknown in the first place but, in the words of Caruth (1996), “gets back to haunt the victim of trauma later on.” The unknown or “apparently unharmed” part of the painful incident distracts the survivor from expressing trauma and makes him familiar with known trauma by the repetition of recurring thoughts-that is, “the traumatic neuroses of war” (p. 61). So, it is hard to understand the trauma mentally because the shock that happened is buried in the memory. It comes back in the form of flashbacks and hallucinations, and it constantly teases the victim and makes a lot of changes in the mind of a survivor.

By extending the idea of known and unknown trauma with respect to discriminative events, Maria P. P. Root’s concept of “insidious trauma,” Brown (1991) maintains that “traumatic effects of oppression that are not necessarily overtly violent or threatening to bodily wellbeing at the given moment, but that do violence to the soul and spirit.” Here, insidious trauma qualifies and supports the concept of Balaev and Caruth, who claim that trauma is hidden but passed to another generation. Hence, trauma is a silence that kills or haunts the victim continuously, and the painful experiences are unable to be fully described by the victims. The victim desires to end his life and feels like a dead person in which death becomes a strong driver that represents the victim’s inner fear and insecurity (Herman, 1992).

According to Caruth (1996), a traumatic event has a double wound where a victim with a painful experience faces trauma two times; one is as a witness to his trauma and the second is that of others. Another psychologist, Laub and Podell (1995), contributed to the idea of witnessing by pointing out that it exists on three levels. He maintains that “witness to oneself” is the first level of witnessing and experiencing the event directly, whereas the second level of witnessing requires the testimony of interviewers, and the third level of witnessing involves the testimony process in which the witnessing can either be live or experienced *via* video or audio recording. These three levels of witnessing are all important to consider if one is to grasp the true nature of trauma.

Likewise, the survivor of traumatic events also undergoes dual trauma, i.e., the painful experience of death firsthand witnessed (known) and the trauma of surviving from death (unknown) and suffers from anxiety, depression, and nightmares, which eventually explains the “real story” that Hwangbo (2004) calls “episodic” memory as opposed to the more general “semantic” memory. Caruth goes on to say that those who have witnessed dreadful, horrifying scenes of violence, or attacks cannot forget these “indelible” memories, and such hyper-visual trauma cannot be changed or altered from the unconscious, implying that it will harm both the victim and the next generation. In relation to the experience of a traumatic event and witnessing, Caruth (1996) and Balaev (2008) focus on the traumatic effect on a traumatized person by calling it a “threat (that) is recognized as much by a mind one moment too late” with an intrusion of “nightmares” or “experiences in flashbacks” that disrupts the victims’ regulated and balanced pattern of the world, as is the case of the survivors of the protagonists in the novel.

In this respect, Kardiner (1941), a psychiatrist, observes victim’s reactions toward unknown/unwanted events by “employing language suggestive of his trying to defend himself during a military assault” where victims’ fixation can last a lifetime (Freud, 1961) because victims receive “a shock of a contingent encounter” in terms of flashbacks or nightdreams that is like “a grain of sand” which “ruins the balance of the symbolic universe of the subject” (Žižek, 1989) and are held captive to painful experiences of their distant past.

## Discussion and analysis

### Korean immigrants’ assimilation causes

According to Balaev (2008), trauma does not always necessarily result from collective human or natural calamities resulting from severe actions such as war(s) or tsunamis, but also as a result of extreme reactions within the victims. This causes a wordless fear that separates or destroys (Henry’s) identity through the intergenerational transmission of trauma *via* language, experience, and memory. In the backdrop study of Balaev’s definition, our analysis of this novel shows that Henry’s father was beaten by a robber at his shop and he was unable to say a single word in his defense due to his poor English language skills. Witnessing his father’s humiliation and news of “another cabbie(’s)” death (Lee, 1995) resonates with Pyke and Dang (2003) words that language and race are the biggest obstacles for Asian–Americans, which, according to Taki (1989), makes Asians “strangers from a different shore.”

Henry’s father refuses to be Korean (Kim, 2009) and realizes that it is “hard to stay Korean” in the United States and chooses “George Washington” (Lee, 1995) as his first and middle name to assimilate into American society. With these pressures to assimilate in mind, George Washington Park encourages Henry to forget his Korean cultural roots and native language and urges him “to casually recite some Shakespeare words,” saying, “I never fathomed the need of the culture even for the smallest acts” (Lee, 1995). George Washington Park hoped that such a demonstration of knowledge of Shakespeare would yield a dual benefit, suggesting an educated elite status, on the one hand, while serving to demonstrate language sophistication on the other. Moreover, Miller (2016) remarks that immigrants develop a false public self at the expense of their native tradition and culture. As it is, in their mind, assimilation into American culture is only completed when the division of us vs. them disappears.

By relying on Balaev’s theory of unspeakable, the intergenerational transmission of trauma is *via* language, experience, and memory (Balaev, 2008) that transfers and reshapes the identity of the next generation, which is clear from the experience of Henry’s father and Henry’s childhood bullied memory. The textual analysis of the novel’s text proves that Henry recalls how–in his childhood–his father, with “halting, polite English,” full of “crash and bang and stop,” had confronted the parent of the white kid and said “my son […] is no good for friends” instead of demanding an apology (Lee, 1995). Henry realizes that his father embodies a person who has learned that to survive, one must silently endure humiliation. Henry also demonstrates the attitude and experience of suffering of his father-that are transferred to Henry in the following words:

He established a pattern for us (family), a timetable. I also wondered whether he was suffering from pain inside, if he, like me, sobbed from time to time for unexplained reasons. I recall sitting with him at a restaurant, both of us eating without savor, uncomfortably (Lee, 1995).

The sufferings of his father, along with the diverse conditions of other migrants, compel Henry to remark that the incessant clamor (in American society) is that one has to forget about his own identity to stay in America; either you belong here, or you must go back (Lee, 1995). Thus, he represses his cultural values, and native language and struggles to assimilate into American culture.

### Dysphasia: Unspeakable as a language effects

The theory of the unspeakable (Balaev, 2008, 2012) establishes the essentialist concept of identity organized around a notion of the “intergenerational sharing of loss and suffering because the actual event is transmitted to descendants.” Like his father, Henry tries to hide his Asian identity by speaking English and adopting a kind of “serial identity” (Lee, 1995), which (Antokoletz Juana, 1993) calls the “false self” that Asian people use to adjust to or make their place in the new community. Consequently, Henry abandons his native tongue for a language he must constantly struggle with and says, “what I know is that America is not so open” (Lee, 1995) that cultural critic Chow (1993) asserts that “individuals become ethnic” as a result of the pressures imposed by societal institutions. Ironically, the United States welcomes newcomers but requires them to speak English rather than accept their native language, thereby assimilating them into American culture.

By taking the views of Michelle Balaev on the unspeakable trope concerning identity, Caruth (1996) extends the idea of intergenerational unspeakability that should not only be applied in a neurobiological sense and to individual identity but also viewed from the perspective of “cultural values.” The analysis of the novel reveals that, like other children of immigrants, Henry “always makes (s) bad errors in speech” (Lee, 1995) and is “half afraid of disappointing” a Korean waitress with a stutter of poorly accented phrases until he finally stops (Lee, 1995). As a consequence, he loses much of his culture and fluency in expressing himself, which Harvey (2002) states as the fundamental “loss.” In the novel, how this loss of Henry’s native language concerning his cultural identity and unspeakability impacts his life negatively is evident when he sees an immigrant worker begs the police officer, “no trouble, no trouble, he will say, shouting it, bowing, shaking his hands, […] breathing it out like a necessary song: no trouble” (Lee, 1995). Henry witnessed this incident that psychologists (Laub and Podell, 1995) call “witness to oneself” and remarked with a sense of shame that the immigrants’ children know this song and “they have learned it well” (Lee, 1995). The sufferings of his father, along with the diverse conditions of other migrants, compel Henry to remark that the incessant clamor (in American society) is that one has to forget about his own identity to stay in America; either you belong here, or you must go back. Thus, he represses his cultural values and native language and struggles to assimilate into American culture.

Chang-Rae Lee succeeds in the novel in dramatizing the problem of sharing and passing along the immigrants’ native tongue intergenerationally, referred to as the “last language” (Lee, 1995). Moreover, the psychologist Antokoletz Juana (1993) supports the opinion of Chang-Rae Lee and compares immigrants’ fear of losing their language and culture with Winnicott’s (1965) idea of “holding a foreign environment” because immigrants are forced to do this to protect themselves. This idea of losing language is also present in the work of the philosopher Hannah Arendt, who says in her poem, *We Refugees*, “we lost our language” under the oppression of natives. In addition to lost language, Derrida (1998) says, “I (immigrant) only have one language; it is not mine.” Lacking their voice, Henry, like other Asian immigrants, always remains under the dominance of White people because they have the advantage of having their voice, including freedom of speech. Lacking power and having no such cultural voice puts Henry at a significant disadvantage. Despite his Korean background, Henry tries to take his place in American society using the English language, making his personality more difficult and complex.

In addition to false identity and loss of cultural values, the intergenerational transfer problem of unspeakability at a domestic level, remembering (in Balaev’s word “memory”) and comparing his childhood language pronunciation with his adulthood linguistic issues (“experience”) at the workplace only causes Henry’s anxiety to increase. Regarding traumatic memory and experience, Gordon (2008) says that trauma “appears dead but is nonetheless powerfully alive.” It looks like it is in the past, yet it is very much present. The vivid memories of Henry’s harrowing experience at a young age with his English classmates who used derogatory remarks taunted him like “yo, China boy, what you doin’ there, practicing,” called him “marble mouth” and other immigrant children the “school retards, the mentals, and the losers” (Lee, 1995) that Ludwig (2007) says the “way you speak defines you” contributed to his trauma of unspeakable.

Henry faces unspeakability with respect to pronunciation issues in his matrimonial life and his white decedent wife (Lelia) comments on how “you said Leel-bya so deliberately” (Lee, 1995), which Balaev calls the “speechless terror” of trauma, a term derived from der Kolk et al. (2012) book, *Traumatic Stress: The Effects of Overwhelming Experience on the Mind, Body, and Society*, which refers to the temporal-linguistic gap induced by the experience. Concerning language problems and experiences, the extent to which a language and its pronunciation are interlinked with each other is concerning. Henry also remarks to himself:

I assumed English would be a simplified version of Korean. You may wear it as another type of coat. I had no idea what a linguistic difference meant back then. Although native speakers may not realize it, English is a scabrous mouthful. There are no distinct sounds for L and R in Korean; the sound is single and lacks a baroque Spanish trill or roll. I will constantly make grammatical mistakes in my speech […]. I will still say riddle for little or bent for vent, but without any emphasis (Lee, 1995).

Henry tries to get rid of the trauma of unspeakability and goes one step ahead from pronunciation to proficiency in colloquial English, which (Horney, 2013) calls “basic anxiety.” Henry realizes that proficiency is also essential, as, without that, “there is no fair way for us to fight” (Lee, 1995), with any white person. Kim (2015) on the deplorable condition of Henry says that Henry suffers from trauma due to “the tensions between the desire for belonging and success,” whereas Lelia also observes that Henry “look(s) like someone listening to himself,” and is not confident enough to utter the right words at the right time in front of native speakers. He can neither express his personality in English nor in Korean and says, “I always hear myself displacing the two languages, conflating them-maybe conflagrating them-for there is so much rubbing and friction, a fire always threatens to blow up between the tongues” (Lee, 1995). As a result, like Shakespeare’s Hamlet, Henry indulges in the nebulous state of “to be or not to be” and says that even in Korean, “the most minor speech seemed trying” in terms of expressing his personality, which (Eng et al., 2003) argues:

It is a double loss to be estranged from one’s native and foreign language. Learning a new language (English) should be viewed as tremendous cognitive growth; the accompanying emotional pain caused by the loss of a previously secure, caring, and familiar language is frequently overlooked or understated.

The unspeakable transgenerational theory of Balaev indicates that individual trauma may pass from one person to another having the same “social or biologic similarities” (Balaev, 2008). Our analysis of the novel reveals that Henry is unable to utter the phrase “I love you” and “never felt comfortable with the phrase,” like his father even though he struggled with it in all of its variations and realized that he “might handicap” Mitt who began to learn English the way he was handicapped by his father, at which, Lelia called Henry an “emotional alien” (Lee, 1995). Furthermore, his attempt to marry Lelia proves as problematic as his effort to assimilate by learning the English language. As a result, neither assimilation nor marrying Lelia help Henry find his place in a White-dominated society or alleviate his anxiety and trauma.

Traumatic events, according to Balaev (2008), “disrupt attachments with self and others.” Chang-Rae Lee qualifies this definition of trauma and shows how the insulting remarks and discrimination succeed in altering Henry’s life entirely, and consequently, a traumatized Henry remarks, “Call me what you will.” An assimilate, a lackey. A duteous foreign-faced boy “I have already been every version of the newcomer who is always fearful, bitter, and sad, whatever you can say or imagine” (Lee, 1995). As Balaev (Herman, 1992) says, the ongoing traumatic events lead to a “contaminated identity” for the protagonist and result in the breakdown of his self-trust. Thus, Henry finds himself condemned to inhabit the lowest level of society and suffers from continuous psychological effects. These long-lasting effects become clear when he stops speaking after realizing that no matter how much he struggles to assimilate, he can never make natives happy despite his best efforts, which marks a border between us vs. him. Similarly, Kim (2016) remarks, “Korean Americans cannot become American without the dying of han.”^1^ Thus, psychological effects lead Henry from the unspeakable trauma into the more profound isolation characteristic of the *trauma of silence*.

### Aphasia: Silence as traumatic effect of unspeakability

A variant of Balaev’s theory, Trauma of the Unspeakable as “silence,” also plays another means of identifying horrible traumatic experiences and suffering from them. Henry’s silent suffering is evident when he says, “suffering is the noblest art, the quieter the better.” According to Laub and Podell (1995), the loss of trust in the external world gives birth to trauma, and a traumatized person ceases to desire a conversation with “the other” and retreats into his inner world of himself. This is the case of Henry, who gradually stops speaking and prefers to remain silent to save himself from embarrassment.

Moreover, Caruth agrees with Balaev on this point that intergenerational silence is also employed as a rhetorical device to demonstrate that it is “speechless fright that destroys the identity” of the victim (Balaev, 2008). Henry’s difficulties with language and his descent into silence prove and qualify this definition, which is also evident from Kwang’s long history of a political career and “mysterious dubbing” speech quality that makes him other in the eyes of Americans who chant slogans such as “America for Americans” (Lee, 1995). As a result of speechless fright, Henry feels he has no choice but to suffer silently and accept his permanent status/identity as someone who breaks or tears up his self-narrative or the social fabric of his society, and the anger caused by such injuries is beyond his ability to control. He finds expression in violent acts, disassociation of self, or unemotional deadening of self-owing to trauma.

In the backdrop study of Balaev’s theory, “identity is formed by the intergenerational transmission of trauma” that creates a “speechless terror.” By applying such terror to silence, the analysis of the novel’s text shows greater suspense and repulsion that allows the reader to imagine Henry’s fear and shattered identity of his son Mitt, like Henry. The White natives put dust in Mitt’s mouth and he experiences racial slurs like “Charlie Chan, face as flat as a pan,” and he boasts to his grandfather at home that he has endured humiliation silently without even crying. From this incident, it is clear how language barriers–as regards the elders’ silence-are contributing to Mitt’s silent suffering, causing him to hide his emotions and bear every insult silently as his elders did. As Balaev (2008) points out:

“Since traumatic experience is intergenerationally transmitted based on shared social characteristics, then everyone can experience trauma through vicarious means based on one’s ethnic, racial, gender, sexual, or economic background.”

The theory shows that the trauma of silence is transmittable within people who “share social or biologic similarities” and establishes the essentialist concept of identity organized around a notion of the “intergenerational sharing of loss and suffering” because the actual event is transmitted to descendants of the same racial members. By relying heavily on the trauma theory of Balaev, the textual analysis proves that Mitt’s death at the hands of White friends who push him down on the ground during the “stupid dog pile” game symbolizes dominant White society and culture. Although Mitt is much better than his father linguistically, in Jack’s words, “no one [……] had ever looked like that,” but Mitt also remains silent like his elders due to their sharing of “social or biologic similarities” (Balaev, 2008). Thus, Mitt has no way to win the game against White people. As Palumbo-Liu (1994) points out, Asian–Americans are always considered hyphenated people, i.e., Asian–American linguistically. According to the analysis, Mitt, like Henry and George Washington Park but in the opposite direction, has adopted the method of suppressing his emotions and feelings like an elder and has lost control of his English language.

Following the tragic event with his grandson, Henry’s father gradually slips deeper into silence and into a state of melancholy, which has a “negative impact on health and psychopathology” and finally leads to a “massive global stroke […] and finally kills him” (Lee, 1995; van der Kolk et al., 2012). This event seems almost inevitable, considering his father’s life-long habit of suppressing his emotions and hiding any signs of weakness as Ah-juh-ma,^2^ acts as “some kind of zombie” and dies from “pneumonia” instead of speaking and sharing.

Of course, the death of Henry’s father and Mitt is a significant shock and brings to head the psychological distress of extreme “emotional labor.” Thus, Henry experiences intergenerational trauma as he has learned from his father, by suppressing his feelings, at which Grinberg and Grinberg (2004) say immigrants postpone their mourning to deal with the external world, a situation that results in a “latency period,” with the mourning postponed for a long time and eventually appearing in the next generation. Similarly, Yoo and Kim (2014) posit that the catalyst that brings the suffering is pushing Henry into the “silent mode of suffering” that characterizes the remainder of Henry’s life. As pointed out earlier, this suffering has been simmering in Henry’s life since childhood on a personal level and also exists for all immigrants on a larger social scale.

### Parasomnia: Effects of silence on mind

The “silent suffering” of Henry causes him to suffer from bad dreams^3^ that reflect the melancholia caused by his trauma. Henry sees an invisible brother in his dreams who can freely express Henry’s feelings, thus serving as an outlet for Henry’s grief. This invisible brother is better at speaking English and is proficient in martial arts, as Mitt was. In addition to this, he is socially fit and “spoke a singing beautiful English.” Another critical point regarding Henry’s mental state is that his “invisible brother” is not an imaginary character for Henry but a person that White people do not want to see in person or in reality. Being unseen, the “invisible brother” serves as a meaningful symbol of how Mitt is ignored or unseen by White people. Henry could have benefited from Mitt’s recuperation from language trauma, but White people chose to ignore him. As a result, Mitt, who could have served as a go-between for Henry and the white world, is tragically killed by them, putting an end to even this surrogate hope. Consequently, Henry can no longer function effectively in his daily life, and he feels even more depressed and traumatized. The psychologists Eng et al. (2003) explain the condition of the traumatized person in the following lines:

The melancholy (i.e., Henry) can keep it (i.e., Mitt) by identifying with the lost thing, but only as a haunting, ghostly identification. It means that the melancholy Henry adopts the vacuity of the lost item or ideal, recognizes that vacuity, and takes part in his self-denigration and destruction of self-esteem.

### Recuperation from trauma

According to Balaev (2008), once the trauma is “spoken” and passed to another, it no longer remains unspeakable, and thus, no longer “traumatic” according to the model’s definition. Finally, to recover, Henry “must place these traumatic experiences into a narrative to be told to others because the memory of these traumatic events is understood as a literal and fixed record of experience stored in an area of the brain that can be unlocked by talking.” Similarly, Hamid (2011) says “silence kills hope. It kills optimism.” Conclusively, Henry visits Emile Luzan, a psychoanalyst by profession but a Pilipino by race. Luzan listens to Henry in detail and comments to Henry, “you will be yourself again, I promise.” Initially, Henry starts to elaborate on his past, and it was a pervasive “story.” Nevertheless, the more Henry attends his sessions, the more he finds solace and feels better. He starts to realize a change in his personality. I “felt stronger anyway [……] because of his kindness and efforts” (Lee, 1995), since Luzan is listening to his inner thoughts. Henry describes his feelings for Luzan in the following lines:

I did not extrapolate; instead, I looped it in the core, spoke openly about my life, and abruptly violated the trust of my father, mother, and wife. I even talked about my dead son. I became dangerously frank, crazy incoherently. I completely stopped listening to him. He let me continue as a good doctor, and at times I thought that he was the only one in the world who could console me. I started to like him honestly (Lee, 1995).

As language and expression are the means to accomplish this, these are the elements Henry will need to work with if he wishes to recover and realize a stable identity. In her famous book, *The Woman Warrior*, Maxine Hong Kingston presents the situation of a schoolgirl who, like Henry, does not speak but keeps silent. In this case, the schoolgirl can accept her own identity by speaking to her reflection in a mirror. Once Henry does this, he can finally reconcile with his past and start working with his wife when “she finally comes back” from the trip and reunites with Henry. At the novel’s end, Henry is on the road to recovery and able to help Lelia in her language center, but instead of any speech work, this time, Lelia decides that Henry will be of more value by telling stories that will give an important message to her students, assuring them that “there is nothing to fear” because “it is fine to mess it all up” (Lee, 1995). In this manner, the children discover that, though English may be challenging to learn and speak, its acquisition symbolizes a metaphorical beast, and the beast can be subdued.

## Conclusion

This research concludes and leaves a message with multiple layers of implications, concepts, and solutions related to the concept of racism and language trauma. For increased exposure in western culture, Korean Americans can start civil rights campaigns like Black Lives Matter, which will also help to demolish a shared adversary: White supremacy. In addition, educational policies may address the issues of racism and language at an earlier age about the richness, variety, and complexity of Asian–American history. The awareness and study of immigrants’ lived experiences and contributions to American culture not only at the regional but national level will help to tackle these issues.

Although collective trauma can stir up the feeling of powerlessness among immigrants and it is toilsome to eradicate all its impacts, the intensity of this trauma can be reduced by establishing certain footings. The political legacy of the host country needs to stretch the welcoming arms to comfort these traumatized masses by launching immigrant-friendly policies such as protection of their basic rights. Another powerful tool to exterminate the effects of collective trauma is digital and print media. Instead of playing its role in fueling the ashes of racism and discrimination, it should promote the art of acceptance of others by extending an olive branch to immigrants who leave behind their homes and share collective fears in the host country.

In the field of minority literature, hence, this research (based on psychoanalytical theory) will pave the way for and open the new door to do more research on the subject matter of language trauma and racism concerning cultural trauma by linking with the cultural traumatic theory of Smelser and Alexander, who illustrate that trauma happens due to an event and always has a historical background. The historical background effects victims intergenerationally and transfers over the span of time. The most important implications of trauma theory for dealing with trauma victims who inherit a traumatic past have not received much attention in Western society.

The first but most important step toward achieving the goal of bringing immutability to the periphery of society is trauma recovery. Restoring trauma exposure (as in Henry’s case) helps victims overcome the detrimental power of trauma, which is associated with recovery from recurrent pain that increases over time. Repeating the trauma exercise through dialogue helps traumatized people overcome the destructive power of trauma. This process allows the traumatic effect of the event to be removed again and again. In this regard, it is important to note that trauma may be repeated in the lives of survivors of past trauma. Traumatic events are not repeated with the same intensity as Spivak (2012) states. “what is said to return is not the repressed but a version of it; the repressed is not the thing that we return intact.” It also breaks the cycle of chronic traumatic shock and heals the emotional wounds of the victims.

The traumatized victim recuperates from trauma by recalling or going through past events repeatedly. So, articulation becomes a source of catharsis for victims. The main idea behind this ideology of catharsis is latency: the trauma is not forgotten but repeated *via* inherited experience to make a person normal. This concept of latency implies a deferral to perception and the assimilation of occasions into the mind’s memory. Furthermore, the latency of experience is also responsible for redundancy, the second significant idea attached to the concept of trauma injury. This insight hypothesis is the emergence of hope in the stories of destruction. The trauma not only creates the problem of destruction but also acts as a healer to bring the victim back to normal life.

## Data availability statement

The original contributions presented in this study are included in the article/supplementary material, further inquiries can be directed to the corresponding authors.

## Author contributions

All authors listed have made a substantial, direct, and intellectual contribution to the work, and approved it for publication.

## Footnotes

1 The word han can be translated as’ an anger or sorrow that arises within a person due to oppression’.

2 Ah-juh-ma (아줌마) is not a name but a general term which means an elderly married Korean woman, just as Ma’am in the United States.

3 Caruth (1996, 61) compared trauma to a recurring nightmare during sleep, which produces a fear-based physical response even after waking up: Dreams that involve memories of traumatic experiences return patients to the psychological state induced by the accident, a circumstance from which they awaken in dismay. In such situations, the dreamer encounters a person who is struggling for life, and in the dream state, that person exists as if in reality, whether still living or deceased.
